# The Differences and Similarities between Allergists and Non-Allergists for Penicillin Allergy Management

**DOI:** 10.1155/2014/214183

**Published:** 2014-02-24

**Authors:** Nayot Suetrong, Jettanong Klaewsongkram

**Affiliations:** ^1^Department of Medicine, Faculty of Medicine, Chulalongkorn University, Bangkok 10330, Thailand; ^2^Division of Allergy and Clinical Immunology, Department of Medicine, Faculty of Medicine, and Allergy and Clinical Immunology Research Group, Chulalongkorn University, Bangkok 10330, Thailand

## Abstract

The purpose of this study was to compare the management of patients with a history of penicillin allergy between allergists and non-allergists in Thailand. A questionnaire was distributed to Thai physicians by online survey. The answers from 205 physicians were analyzed. The discrepancy of penicillin allergy management between allergists and non-allergists was clearly demonstrated in patients with a history of an immediate reaction in the presence of penicillin skin test (*P* < 0.01) and in patients with a history of Stevens-Johnson syndrome (*P* < 0.05) from penicillin. Allergists are more willing to confirm penicillin allergic status, more likely to carefully administer penicillin even after negative skin test, but less concerned for the potential cross-reactivity with 3rd and 4th generation cephalosporins, compared to non-allergists. The lack of penicillin skin test reagents, the reliability of penicillin allergy history, and medicolegal problem were the main reasons for prescribing alternate antibiotics without confirmation of penicillin allergic status. In summary, the different management of penicillin allergy between allergists and non-allergists was significantly demonstrated in patients with a history of severe non-immediate reaction and in patients with a history of an immediate reaction when a penicillin skin test is available.

## 1. Introduction

Penicillin allergy is one of the most commonly reported drug allergies worldwide. About 10% of the general population report suspected allergic reactions to penicillin [1]. However, only a minority of patients with such a history are actually allergic to penicillin(s), based on the results of allergological work-up [2]. In real life, most physicians prefer to prescribe alternate antibiotics to these patients, unless a penicillin allergic status can be excluded [3, 4]. Since the prescription of alternate antibiotics may have undesirable consequences in terms of antibiotic susceptibility, adverse reactions, and health economics, the confirmation of penicillin allergic status would be beneficial in patients with a suspected history of penicillin allergy.

In clinical practice, the confirmation of penicillin allergic status is not always feasible, which results in unnecessary avoidance of beta-lactam antibiotics in patients who are overdiagnosed [5]. It is well recognized nowadays that the cross-reactivity rate among beta-lactams is lower than previously expected [6]. However, the consensus recommendation of antibiotic selection in these “suspected” penicillin allergic patients has yet to be established [7–9]. Allergists, physicians specialized in managing allergy and immunology disorders, are responsible for confirming penicillin allergic status, preventing overdiagnosis, and determining appropriate alternative antibiotics. In reality, the number of certified allergists in many countries may not be sufficient to manage this common problem. This task is particularly difficult in Thailand, as there are only 148 certified allergists (28 adult allergists and 120 pediatric allergists), among a total of 45,124 medical doctors, or an equivalent of 0.3% registered medical doctors in the country [10]. As a result, the selection of antibiotics in these patients is often decided by doctors of other specialties in real life before they can seek help from allergists. Even among Thai allergists themselves, penicillin skin test is mainly performed by using penicillin G alone and the use of penicilloyl-polylysine and minor determinant mixture is mostly limited for research purposes. The confirmation of drug allergy status has not been emphasized in medical school curriculum resulting in inadequate knowledge for non-allergists to handle this problem. The potential role of pharmacists in conducting penicillin skin testing in these patients has also been suggested but rarely implemented [11].

It is understandable that doctors in other specialties may have limited knowledge and different perspectives about managing allergic disorders, as compared to allergists [12]. Their perspectives and fundamental knowledge on penicillin allergy might have an impact on how they manage these patients in clinical practice.

The lack of allergists and standard skin test reagents for the diagnosis of penicillin allergy makes the confirmation of penicillin allergic status not always possible [13–17]. Types of medical practice settings and clinical practice duration may also influence the selection of antibiotics in patients with a history of penicillin allergy. The selection of antibiotic prescription in patients with different types of previous penicillin reactions could be influenced by several factors such as the availability of skin test reagents and medicolegal risk. It would be interesting to know how patients with a history of penicillin allergy are mainly managed in the real life by non-allergists where certified allergists may not always be available.

The purpose of this study was to comparatively survey the management of penicillin allergy between allergists and non-allergists in Thailand in patients with different patterns of penicillin-induced suspected allergic reactions. The awareness of cross-reactivity among beta-lactam antibiotics as well as the knowledge and attitudes towards penicillin allergy management was also determined. The impacts of areas of expertise, types of medical practice settings, and duration of clinical practice on penicillin allergy management were also analyzed.

## 2. Methods and Materials

This study was a cross-sectional survey on the management of patients with a suspected history of penicillin allergy in Thailand. After approval by the Ethics and Research Committee of Faculty of Medicine at Chulalongkorn University, the web-based questionnaire was created by using Google Docs and e-mailed to 1,000 Thai physicians (57 allergists and 943 non-allergists in various fields) throughout the country. The questionnaire was focused on four aspects: (1) the management of patients with a history of penicillin-induced immediate reactions in the presence and absence of penicillin skin test reagents, (2) the management of patients with a history of penicillin-induced non-immediate reactions, (3) the prescription of other beta-lactams in patients with a history of penicillin allergy, and (4) the fundamental knowledge of penicillin allergy skin testing and attitudes towards the management of patients with a history of penicillin allergy in Thailand. Details of the web-based questionnaire are available in Supplemental Appendix Ain Supplementary Material available online at http://dx.doi.org/10.1155/2014/214183.

The online survey responses were automatically collected and subsequently analyzed using SPSS 17.0 for Windows (SPSS, Chicago, Il, USA). The actual rates of penicillin allergy in patients with a suspected history estimated by physicians were calculated by using class interval arithmetic means. Chi-square test and multinomial logistic regression were used for univariate and multivariate analysis. *P* values < 0.05 were considered statistically significant.

## 3. Results

### 3.1. Characteristics of Thai Physicians Participating in This Survey

A total of 205 completed surveys were received (a 20.5% response rate) from the online questionnaire. 14.1% were adult or pediatric allergists, while 26.3%, 40.5%, and 19.0% were general practitioners, internists or pediatricians (excluding allergists), and other specialists, respectively. Almost half (45.9%) of the responders worked for an academic institute (university hospital or research center) and 19.0% worked in a primary care hospital, 22.9% in a general/provincial hospital, and 12.2% in the private practice sector. About 45.9% of the responders have less than 5-year experience in clinical practice, while 30.2% and 23.9% of them have 5–10 years and more than 10 years of practice experience, respectively (Table 1).

### 3.2. The Management of Patients with a History of Penicillin-Induced Immediate Reaction according to the Availability and Result of Penicillin Skin Testing

**Figure 1:**
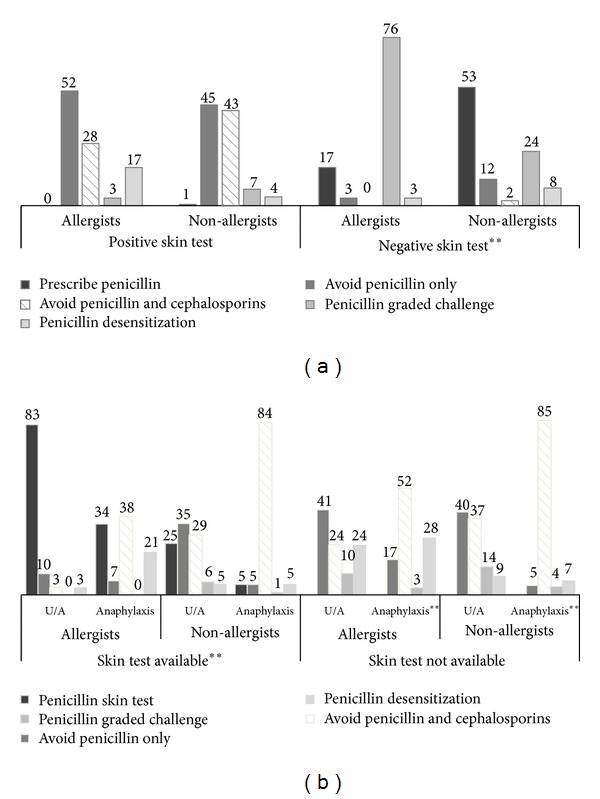
Management of patients with a history of penicillin-induced immediate reaction. The difference in penicillin allergy management between patients with mild and severe immediate allergic reactions was clearly demonstrated regardless of skin test availability and depending on penicillin skin test results (data represent percentages of each group, ∗∗ represents *P* values < 0.01 between allergists and non-allergists, and U/A: urticaria and/or angioedema).

When penicillin administration was indicated, the different management between allergists and non-allergists in patients with a history of penicillin-induced immediate reaction was not statistically different if skin testing was positive (*P* value = 0.06) but was clearly demonstrated if the penicillin skin test was negative (*P* value < 0.01) (Figure 1(a)). 79.3% of allergists and 88.1% of non-allergists would avoid penicillin or both penicillin and cephalosporins if penicillin skin testing yielded a positive result. In contrast, 75.9% of allergists would use graded challenge first if penicillin skin test was negative, while 53.4% of non-allergists would prescribe penicillin normally.

A question was addressed whether the severity of the immediate reaction influenced patient management in the presence or absence of penicillin skin test reagents (Figure 1(b)). The difference of decision making between allergists and non-allergists was observed in patients for whom penicillin was indicated regardless of clinical severity and who had been skin-tested with penicillin reagents (*P* values < 0.01). If a penicillin skin test was not available, the management of patients with a history of penicillin-induced severe immediate reaction (anaphylaxis) between allergists and non-allergists was still different (*P* value < 0.01), but no longer different (*P* value = 0.12) in patients with a history of penicillin-induced mild immediate reaction (urticaria and/or angioedema).

In patients with a history of anaphylaxis, 83.5–84.7% of non-allergists would avoid both penicillin and cephalosporins regardless of skin testing availability. In contrast, 34.5% of allergists would perform a penicillin skin test if available, and 20.7% considered prescribing penicillin using a desensitization technique in patients requiring penicillin therapy. In patients with a history of penicillin-induced urticaria or angioedema, 82.8% of allergists would perform penicillin skin testing first, while only 25.0% of non-allergists would do so. However, if the penicillin skin test was not available, the majority of physicians (65.5% of allergists and 77.3% of non-allergists) would avoid both penicillin and cephalosporins.

### 3.3. The Management of Patients with a History of Penicillin-Induced Non-Immediate Reaction

**Figure 2:**
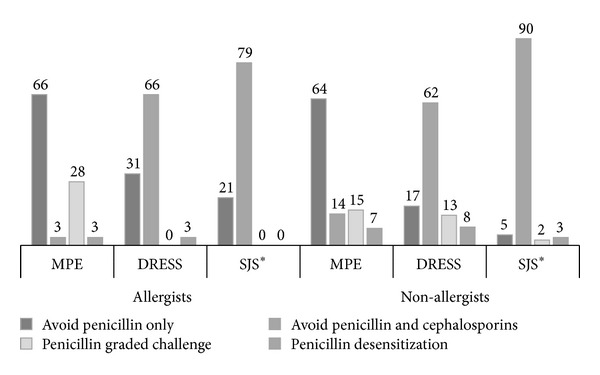
Management of patients with a history of penicillin-induced non-immediate reactions. Antibiotic prescription among Thai physicians in patients with various manifestations of penicillin-induced non-immediate reaction (data represent percentages of each group, ∗ represents *P* value < 0.05 between allergists and non-allergists, MPE: maculopapular exanthema, DRESS; drug reaction with eosinophilia and systemic symptoms, SJS: Stevens-Johnson syndrome).

Regarding the management of patients with a history of penicillin-induced non-immediate reaction, the prescription pattern between allergists and non-allergists was significantly different in Stevens-Johnson syndrome (SJS, *P* value = 0.03), borderline different in drug reaction with eosinophilia and systemic symptoms (DRESS, *P* value = 0.05), but not statistically different in maculopapular exanthema (MPE, *P* value = 0.20) (Figure 2). Only 3.4% of allergists would readminister penicillin with graded challenge or desensitization methods in patients with a history of DRESS, while 21.1% of non-allergists considered doing so. Some of non-allergists (5.7%) still considered prescribing penicillin with graded challenge or desensitization methods in patients with a history of SJS, while none of allergists would do it. In contrast, the majority of both allergists (65.5%) and non-allergists (63.6%) would avoid only penicillin in patients with a history of penicillin-induced MPE.

### 3.4. Patterns of Beta-Lactam Prescription in Patients with Unconfirmed Allergic Reaction to Penicillin

**Figure 3:**
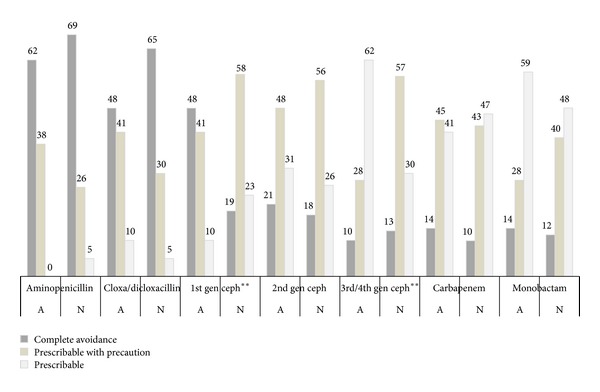
Patterns of beta-lactam prescription in patients with unconfirmed allergic reactions to penicillin. The cross-reactivity between penicillin and other beta-lactams was mainly concerned among Thai physicians when prescribing aminopenicillin and the different decision making between allergists and non-allergists was significantly observed when prescribing first or third/fourth generations of cephalosporins (data represent percentages of each group, ∗∗ represents *P* values < 0.01 between allergists and non-allergists, gen ceph: generations of cephalosporin, A: allergists, and N: non-allergists).

When penicillin skin test reagents are not available, the decision making between allergists and non-allergists in terms of beta-lactam prescription in patients with an unconfirmed history of an immediate reaction to penicillin was different in the case of first generation and third/fourth generation cephalosporins (*P* values < 0.01) but not with other drugs (Figure 3). Aminopenicillins were completely avoided by more than 60% of all Thai physicians, while about half of them considered carbapenem and monobactam “prescribable.” First generation cephalosporins would be completely avoided by 48.3% of allergists or prescribed with precaution by 41.4%. In contrast, only 18.8% of non-allergists would completely avoid these drugs, while 58.0% of non-allergists indicated that they would prescribe them with precaution. For third and fourth generation cephalosporins, 62.1% of allergists said that they could be prescribed, while only 29.5% of non-allergists agreed so.

### 3.5. Knowledge and Attitudes of Thai Physicians towards the Management of Patients with a History of Penicillin Allergy

**Figure 4:**
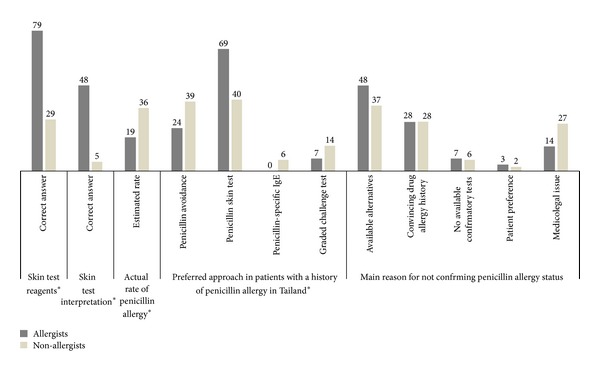
Knowledge and attitudes towards the management of patients with a history of penicillin-induced immediate reactions. Non-allergists have limited knowledge concerning penicillin skin test reagents and interpretation. Different opinions between allergists and non-allergists were demonstrated regarding the reasons for not confirming penicillin allergy status and the preferred approach to confirm penicillin allergy (data represent percentages of each group; ∗ represents *P* values < 0.05 between allergists and non-allergists).

Regarding basic knowledge of penicillin skin testing for diagnosis of an immediate reaction, less than half of non-allergists (29.1%) have accurate knowledge on the appropriate recommended skin test reagents (penicilloyl-polylysine and minor determinants) and only 5.1% of them know how to correctly interpret penicillin skin test results according to the ENDA recommendation (an increase in wheal diameter greater than 3 mm read 15–20 minutes after the test compared to the initial wheal size) [18]. It is worth noting that only 48.3% of trained allergists in Thailand could properly interpret the result of penicillin skin tests as well (Figure 4). The actual rate of penicillin allergy in patients with a suspected history estimated by allergists was significantly lower than that estimated by non-allergists (18.9% to 35.6%, resp., *P* value = 0.02).

A difference in opinion between allergists and non-allergists was observed. The preferred approach to manage patients with a history of an immediate reaction to penicillin was significantly different between allergists and non-allergists (*P* value = 0.04). While the majority of allergists (69.0%) favored penicillin skin tests over penicillin avoidance (24.1%), both approaches were equally elected by the non-allergist group (39.2–40.3% each). The reasons for prescribing an alternate drug without confirming penicillin allergy status were not statistically different between allergists and non-allergists (*P* value = 0.50). The easy availability of alternate antibiotics was the main reason, followed by a convincing drug allergy history. Interestingly, the medicolegal problem was another main concern for Thai physicians, especially among non-allergists.

### 3.6. Multivariate Analysis of Factors Influencing Penicillin Allergy Management among Thai Physicians

**Figure 5:**
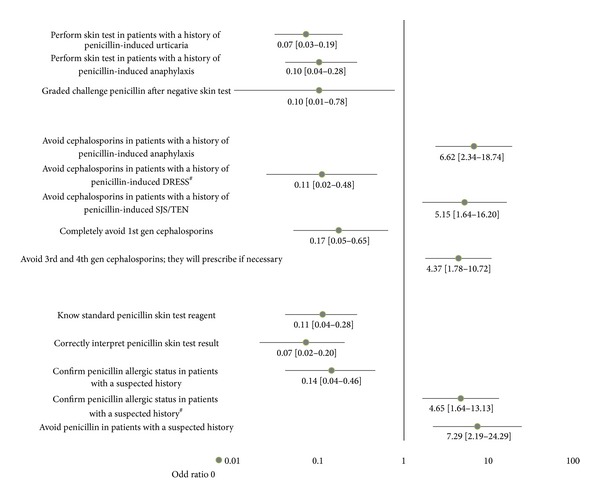
Factors affecting penicillin allergy management. Multivariate analysis by using multinomial logistic regression demonstrates that area of expertise was the main factor determining penicillin allergy management. Data represent the odds ratio with 95% confidence intervals of how non-allergists manage penicillin allergy compared to allergists except #, which represent the odds ratio with 95% confidence intervals of how physicians who have less than 5 years' clinical experience manage penicillin allergy compared to those who have longer than 10 years' clinical experience.

Factors possibly influencing penicillin allergy management were analysed based on physician's area of expertise, medical practice setting, and clinical practice duration. The results indicated that the most important factor determining the management of penicillin allergy was the area of expertise (see Appendix B). After multivariate analysis, penicillin allergy management by allergists was significantly different from non-allergist counterparts (Figure 5).

Non-allergists preferred to confirm an allergic history by means of skin tests in patients with a history of penicillin-induced urticaria and anaphylaxis, much less than allergists would do (0.07- and 0.10-fold, resp.), and less likely to administer penicillin with graded challenge technique (0.10-fold) in patients with a suspected history of penicillin allergy after negative skin test, compared to allergists. In contrast, non-allergists were more likely to avoid not only penicillin but also cephalosporins in patients with a history of penicillin-induced anaphylaxis and SJS than allergists would do so (6.62- and 5.15-fold, resp.). Interestingly, they avoided first generation cephalosporins much less than allergists (0.17-fold) in patients with a history of penicillin allergy. Probably due to limited knowledge in penicillin skin test procedure and interpretation, non-allergists rather preferred to avoid penicillin (7.29 folds) than to confirm allergic status (0.14 fold), as compared to their allergist counterparts.

Clinical practice duration also had some influence since physicians with less than 5 years of experience in practice were more in favor of penicillin allergy confirmation (4.65-fold) than those practicing medicine longer than 10 years and were less likely to avoid cephalosporins in patients with a history of penicillin-induced DRESS (0.11-fold).

## 4. Discussion

Although allergists are primarily responsible for the management of patients with a history of a drug allergy, in the real life, these patients are often cared for by doctors in other specialties due to the shortage of certified allergists in some areas. This study surveyed how the problem of penicillin allergy in different circumstances was managed by allergists and non-allergists, including their knowledge and attitudes towards this problem.

Our study shows that allergists are more willing to confirm the status of penicillin allergy and more inclined to use a desensitization procedure in patients with a history of penicillin-induced immediate reaction, as compared to non-allergists. The study also shows that the limited availability of penicillin skin tests clearly impacts their clinical judgment. If the penicillin skin testing was not available, the discrepancy of patient management between allergists and non-allergists in patients with a history of penicillin-induced urticaria or angioedema would no longer be observed, since allergists also avoided penicillin ± cephalosporins in these cases. Interestingly, the majority of allergists were very cautious when prescribing penicillin in patients with a suspected history even after a negative penicillin skin test, while half of non-allergists would prescribe penicillin normally in a similar circumstance.

Different views on beta-lactam cross-reactivity between allergists and non-allergists were noted. Interestingly, allergists were more reluctant to prescribe first generation cephalosporins in penicillin allergic patients while being less concerned about third/fourth generation cephalosporins, as compared to their non-allergist counterparts. Current data indicates that the potential cross-reactivity with penicillin is noteworthy only in first generation cephalosporins [19]. In terms of non-immediate reactions, more non-allergists considered avoiding cephalosporins in patients with a history of penicillin-induced SJS, as compared to allergists. No statistical difference was observed between allergists and non-allergists in the management of penicillin-induced MPE and DRESS. However, allergists seemed less likely to perform graded challenge or desensitize patients with previous DRESS.

Regarding knowledge and attitudes towards penicillin allergy management, it was clear that non-allergists have limited knowledge regarding penicillin skin test reagents and interpretation as compared to certified allergists. Even though both allergists and non-allergists agreed that majority of patients with a history of penicillin allergy are not truly allergic, the allergists' estimated rate of penicillin allergy in these patients was significantly lower than that of their non-allergist counterparts. Surprisingly, less than half of Thai allergists could correctly interpret penicillin skin test results as well. A refresher course for allergists on drug allergy testing should be organized. The appropriate modalities of allergological work-up in patients with suspected beta-lactam hypersensitivity should be finalized. The lack of penicillin metabolites (penicilloyl-polylysine and minor determinant mixture) could potentially be replaced by the soluble forms of the suspected beta-lactams or other beta-lactams from the same classes, along with benzylpenicillin and aminopenicillin, as skin test reagents since they are more easily available and have good predictive values in clinical practice [20].

While penicillin skin testing was more favoured among allergists, many non-allergists still preferred penicillin avoidance. The availability of alternate drugs and the convincing drug allergy history were the main factors for prescription of alternate antibiotics instead of the confirmation of penicillin allergy status. It is worth mentioning that the medicolegal problem from harmful reactions, which are possible after a drug rechallenge, was one of the major concerns for not confirming the status of penicillin allergy particularly among non-allergists. The launch of a national standard practice guideline to manage patients with a history of penicillin allergy would be helpful to prevent medicolegal problems. Health economics and outcome research regarding the confirmation of penicillin allergy in patients with a suspected history should be carefully investigated.

The study demonstrates the diversity of management in patients with a history of penicillin allergy among Thai physicians, although most of them knew that only a minority of these patients are truly allergic. Although the duration of medical practice may also play a role, the results of our study emphasize that the area of expertise was the most important factor determining penicillin allergy management. As the different opinions between allergists and non-allergists were statistically significant in patients with a history of severe non-immediate reaction and in patients with a history of mild immediate reaction if penicillin skin test reagents are available, we recommend that these patients are managed by allergists. Nevertheless, penicillin skin test reagents should be provided to practice allergists and the accurate skin test procedure emphasized. In contrast, it might be possible that non-allergists should be allowed to handle patients with a history of penicillin-induced mild non-immediate reaction (simple MPE) if no certified allergist is available. According to our study, the current approach between allergists and non-allergists in this patient group is already similar. In fact, graded challenge test is the recommended procedure for any physicians knowledgeable in treating adverse drug reactions to manage these patients, since the risk to develop severe reaction is small [21]. In this regard, updated information about beta-lactam cross-reactivity and drug readministration by graded challenge technique should be provided to non-allergists to minimize patient risk.

There are some limitations to this study. The ratio of non-allergists to allergists was 6 : 1 due to the limited number of allergists in the country. In fact, the number of allergists who replied to this survey was already one-fifth of total certified allergists in Thailand. The response rate was rather low but still within a similar range to prior studies [3, 9]. Data from this study may not represent the views of Thai physicians as a whole since almost half of the responders worked in academia. Although medical practice setting alone did not have a significant impact on penicillin allergy management after multivariate adjustment, stratified survey in all types of medical practice could possibly be conducted to reduce response bias.

## 5. Conclusions

The different management of penicillin allergy between allergists and non-allergists was mainly observed in patients with a history of severe non-immediate reaction and in patients with a history of an immediate reaction, particularly in the patients who have been skin-tested with penicillin reagents. Pitfalls in penicillin allergy management by both allergists and non-allergists are addressed. The possible role of non-allergists in the management of patients with a history of penicillin-induced mild non-immediate reaction has been raised.

## Supplementary Material

The Supplementary Material demonstrates the survey questionnaire about penicillin allergy management emailed to Thai physicians in this study (Appendix A) and factors affecting penicillin allergy management among Thai physicians in different scenarios (Appendix B)

## Figures and Tables

**Table 1:** Characteristics of Thai physicians participating in this survey.

Physicians	Total (*N* = 205)
Area of expertise	
General practitioners	54 (26.3%)
Internists and pediatricians	83 (40.5%)
Allergists	29 (14.1%)
Other specialists	39 (19.0%)
Medical practice settings	
Primary care hospitals	39 (19.0%)
General hospitals/provincial hospitals	47 (22.9%)
Academic institutes	94 (45.9%)
Private practice	25 (12.2%)
Medical practice duration	
Less than 5 years	94 (45.9%)
5–10 years	62 (30.2%)
More than 10 years	49 (23.9%)
