# Supplementary material for: The Differences and Similarities between Allergists and Non-Allergists for Penicillin Allergy Management
Source: J Allergy (Cairo). 2014 Feb 24;2014:214183. doi: 10.1155/2014/214183 (PMC3953625; doi:10.1155/2014/214183)
Supplement: Supplementary file 1 — The Supplementary Material demonstrates the survey questionnaire about penicillin allergy management emailed to Thai physicians in this study (Appendix A) and factors affecting penicillin allergy management among Thai physicians in different scenarios (Appendix B) [file 214183.f1.pdf]

Appendix A.

Survey questionnaire about penicillin allergy management

(Select one best answer in the following clinical scenario questions : MPE = maculopapular exanthema, DRESS = drug reaction with eosinophilia and systemic symptoms, SJS = Stevens-Johnson syndrome)

- Preferred management in patients with a history of penicillin-induced urticaria/angioedema when penicillin is indicated in the presence and absence of penicillin skin test reagent
  a) Perform penicillin skin test
  b) Avoid penicillin
  c) Avoid penicillin and cephalosporins
  d) Perform penicillin graded challenge
  e) Perform penicillin desensitization

- Preferred management in patients with a history of penicillin-induced anaphylaxis when penicillin is indicated in the presence and absence of penicillin skin test reagent
  a) Perform penicillin skin test
  b) Avoid penicillin
  c) Avoid penicillin and cephalosporins
  d) Perform penicillin graded challenge
  e) Perform penicillin desensitization

- Preferred management in patients with a history of penicillin-induced an immediate reaction when penicillin is indicated according to positive and negative penicillin skin test results
  a) Prescribe penicillin normally

b) Avoid penicillin

c) Avoid penicillin and cephalosporins

d) Perform penicillin graded challenge

e) Perform penicillin desensitization

- Preferred management in patients with a history of penicillin-induced non-immediate reactions (MPE, DRESS, or SJS) when penicillin is indicated

a) Avoid penicillin only

b) Avoid penicillin and cephalosporins

c) Perform penicillin graded challenge

d) Perform penicillin desensitization

- Prescription of the following antibiotics in patients with unconfirmed allergic reaction to penicillin

1. Aminopenicillins
2. Cloxa/dicloxacillin
3. 1st generation cephalosporins
4. 2nd generation cephalosporins
5. 3rd & 4th generation cephalosporins
6. Carbapenem
7. Monobactam

a) Complete avoidance

b) Prescribable with precaution

c) Prescribable

- The recommended penicillin skin test reagent (s) for the confirmation of patients with a history of an immediate reaction to penicillin

a) Freshly prepared penicillin G solution

b) Aged penicillin G solution

c) Penicilloyl-polylysine

d) Minor determinant mixtures

e) Penicilloyl-polylysine + minor determinant mixtures [correct answer]

- How to correctly interpret penicillin skin test as a positive intradermal test?

a) Final wheal diameter at least 5 mm

b) Final wheal diameter at least 2 times larger than negative (normal saline) control

c) Final wheal diameter at least 2 times larger than the initial size

d) Final wheal diameter increase at least 3 mm larger than the initial size [correct answer]

- The estimated actual allergic rate in patients with a history of penicillin allergy

a) 0-20%

b) 20-40%

c) 40-60%

d) 60-80%

e) 80-100%

- Main reason for using alternative antibiotics without confirming penicillin allergic status in patients with a suspected history

a) Convincing history

b) No available diagnostic tests

c) Patient preference

d) Availability of alternative drugs

e) Medico-legal issue

- Your opinion for the management of patients with a history of penicillin-induced immediate reaction in Thailand

a) Avoid penicillin

b) Perform penicillin skin test

c) Measure penicillin-specific IgE

d) Perform penicillin graded challenge

Appendix B.

P values of univariate and multivariate analysis of factors affecting penicillin allergy management among Thai physicians in different scenarios (NS = not significant)

**Scenarios of penicillin allergy management**	**Areas of expertise**		**Medical practice settings**		**Medical practice duration**	
	**Univariate**	**Multivariate**	**Univariate**	**Multivariate**	**Univariate**	**Multivariate**
1. Immediate reactions (mild)<br>Unavailable skin test	0.28	NS	0.66	NS	0.66	NS
2. Immediate reactions (severe)<br>Unavailable skin test	**< 0.01**	**< 0.01**	0.12	NS	0.94	NS
3. Immediate reaction (mild)<br>Available skin test	**< 0.01**	**< 0.01**	0.51	NS	**< 0.01**	NS
4. Immediate reaction (severe)<br>Available skin test	**< 0.01**	**< 0.01**	0.30	NS	**< 0.01**	NS
5. Immediate reactions<br>Positive skin test	0.12	NS	0.26	NS	0.38	NS
6. Immediate reactions<br>Negative skin test	**< 0.01**	**< 0.01**	0.21	NS	**0.03**	NS
7. MPE	0.17	NS	0.38	NS	0.23	NS
8. DRESS	0.17	NS	0.30	NS	**< 0.01**	**< 0.01**

9. SJS	**0.05**	**0.05**	0.19	NS	0.18	NS
10. Aminopenicillin	0.19	NS	0.62	NS	0.21	NS
11. Cloxa/dicloxacillin	0.50	NS	0.83	NS	0.59	NS
12. 1st gen cephalosporin	**0.03**	**0.03**	**0.05**	NS	**0.03**	NS
13. 2nd gen cephalosporin	0.95	NS	0.75	NS	0.65	NS
14. 3rd/ 4rh gen cephalosporin	**0.01**	**0.02**	0.10	NS	0.50	NS
15. Carbapenem	0.19	NS	**0.04**	NS	**0.09**	NS
16. Monobactam	0.35	NS	**0.08**	NS	0.40	NS
17. Correct test reagents	**< 0.01**	**< 0.01**	**< 0.01**	NS	**< 0.01**	NS
18. Correct interpretation	**< 0.01**	**< 0.01**	**< 0.01**	NS	0.17	NS
19. Reasons for use alternative drugs without confirm allergic status	0.47	NS	0.99	NS	0.08	NS
21. Preferred recommendation	**< 0.01**	**< 0.01**	0.33	NS	**< 0.01**	**0.02**
